# Supplementary material for: Genome-wide association study of Buruli ulcer in rural Benin highlights role of two LncRNAs and the autophagy pathway
Source: Commun Biol. 2020 Apr 20;3:177. doi: 10.1038/s42003-020-0920-6 (PMC7171125; doi:10.1038/s42003-020-0920-6)
Supplement: Supplementary file 1 — Supplementary Information [file 42003_2020_920_MOESM1_ESM.pdf]

Supplementary Information

Manuscript: “Genome-wide association study of Buruli ulcer in rural Benin highlights role of two LncRNAs and the autophagy pathway”

Supplementary Information includes five Supplementary Figures and one Supplementary Table.

Supplementary Figures

Supplementary Figure 1. Principal component analysis (PCA) to study population stratification.

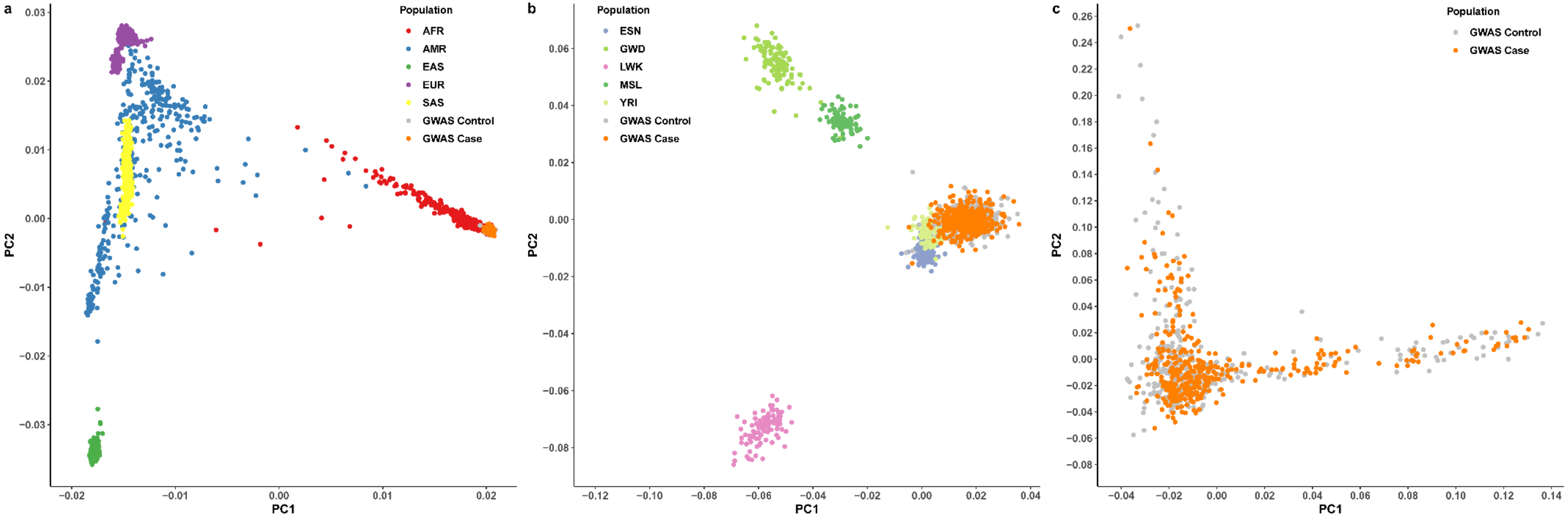

(a) First (*x*-axis) and second (*y*-axis) components of the PCA performed on the populations of the 1000 Genomes Project together with the Buruli Ulcer patients and the Buruli ulcer-free controls used in our GWAS. Both cases and controls cluster with the African populations (AFR: African; AMR: admixed American; EAS: East Asian; EUR: European; SAS: South Asian). (b) First (*x*-axis) and second (*y*-axis) components of the refined PCA performed on the sub-Saharan African populations of the 1000 Genomes Project together with the Buruli ulcer patients and the Buruli ulcer-free controls used in our GWAS. Both cases and controls cluster with the Yoruba in Ibadan, Nigeria (YRI) and the Esan in Nigeria (ESN) populations (GWD: Gambian in western divisions in the Gambia; LWK: Luhya in Webuye, Kenya; MSL: Mende in Sierra Leone). (c) First (x-axis) and second (y-axis) components of the refined PCA performed on the Buruli ulcer patients and the Buruli ulcer-free controls used in our GWAS after excluding one individual from each pair of first and second degree relatives.

Supplementary Figure 2. Quantile-quantile plots of the GWAS for Buruli ulcer.

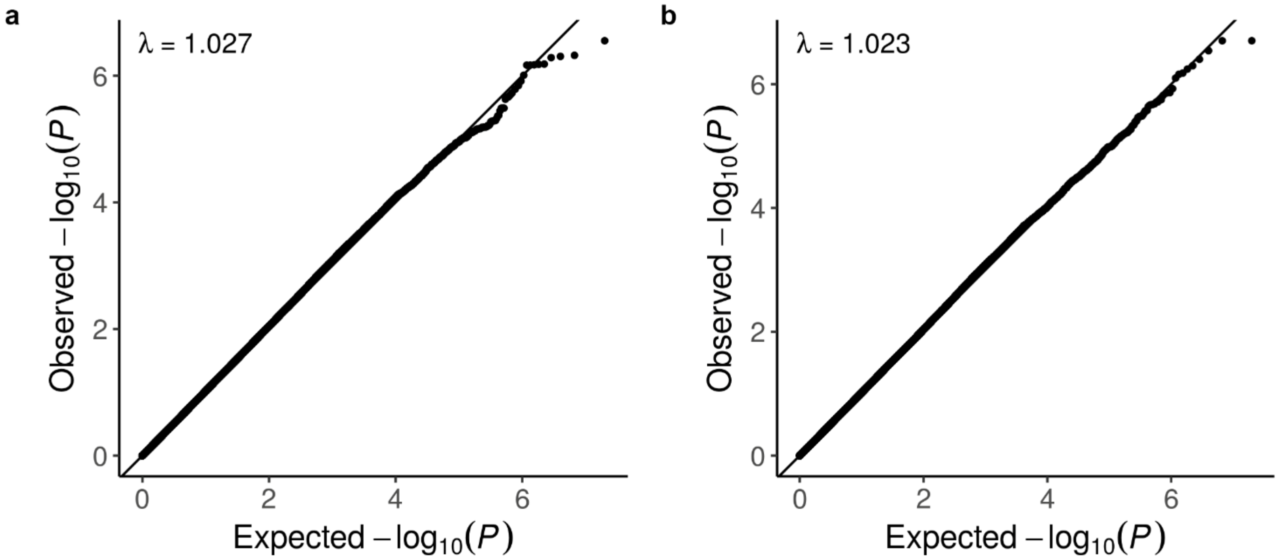

Quantile-quantile plot of genome-wide *P* values for the association between variants and Buruli ulcer, considering either a binary (affected/unaffected; panel A) or a censored (age at onset for Buruli ulcer patients and age at examination for the exposed controls; panel B) phenotype. These analyses include 10,014,109 high-quality genotyped or imputed variants with a minor allele frequency (MAF) above 0.02 and 0.05 respectively, without adjustment, under the additive model. The observed $-\log_{10}$(*P* values) are plotted against expected $-\log_{10}$(*P* values), and were obtained in likelihood ratio tests for the logistic regression (a) and Cox model (b). No significant deviations from expectations were observed. In both cases, the genomic inflation factor (λ) was close to 1.

Supplementary Figure 3. Regional linkage disequilibrium plots after conditioning on the main associated SNPs.

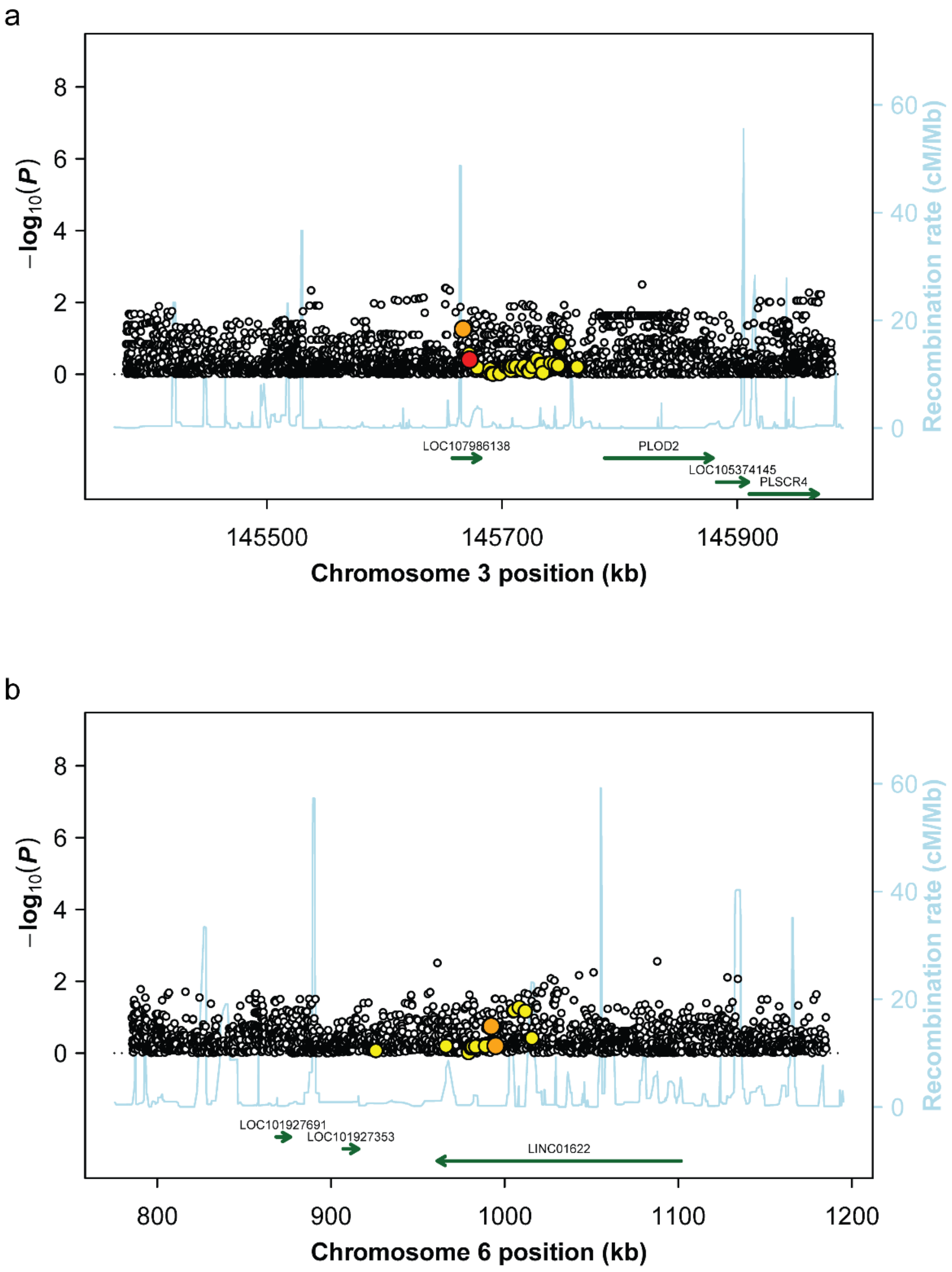

Residual evidence of association between Buruli ulcer and variants located in the vicinity of rs9814705 and rs9814705 according to coordinates in kb as provided in GRCh37 (*x*-axis). Residual evidence of association is expressed as -$\log_{10}(P)$ of the association test after adjustment on rs9814705 (a) and rs9814705 (b). Recombination rates in cM/Mb are also given (Right *y*-axis; light blue line). A color-coded scheme is used to display the LD of the tested variants with the driving SNP (red: $r^2 > 0.8$, orange: $0.5 < r^2 < 0.8$, and yellow: $0.2 < r^2 < 0.5$). Known genes are also provided for each of the chromosomal regions with arrows indicating their orientation.

Supplementary Figure 4. Correlation between *P* values obtained with general linear models *vs.* mixed models.

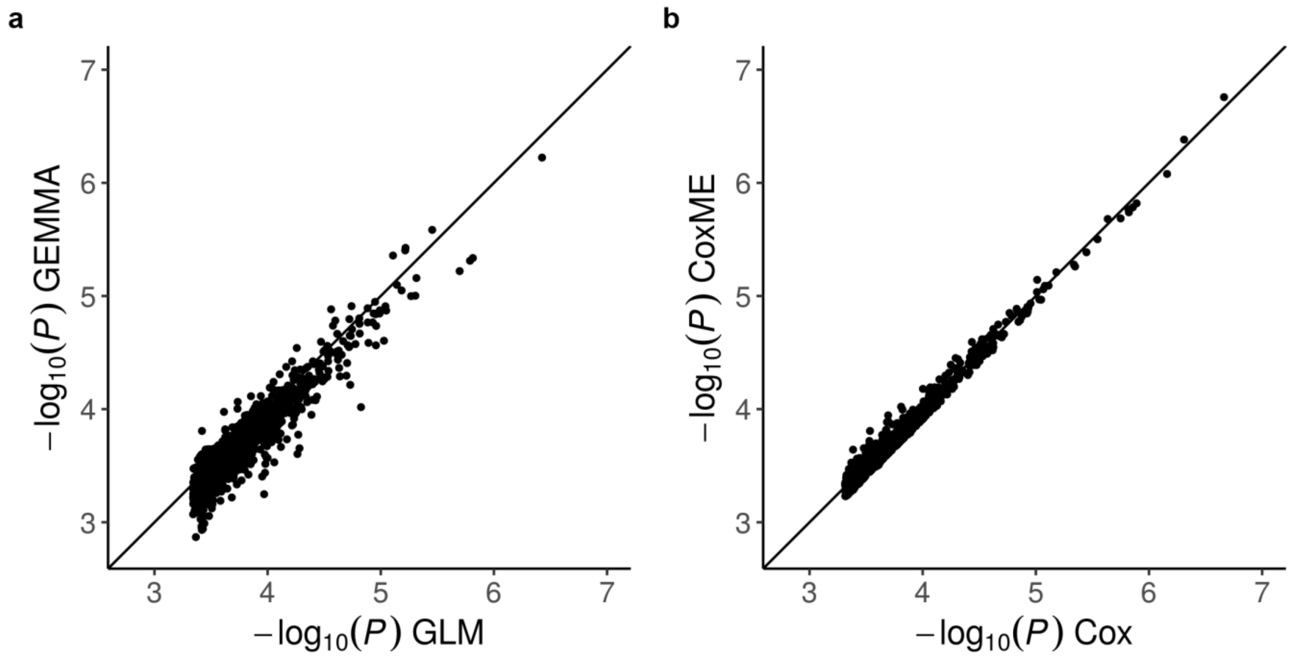

Top 1,000 –$\log_{10}$(*P* values) are plotted for (a) logistic regression: GLM (*x*-axis) *vs*. mixed model (GEMMA) (*y*-axis), r=0.90), and (b) survival analysis: Cox proportional hazards model (*x*-axis) *vs*. mixed-effect coxme (*y*-axis), r=0.97) where *r* is Spearman's correlation coefficient. These findings validate the use of GLM and Cox models in the replication sample.

Supplementary Figure 5. Power estimates for the association study on the discovery sample.

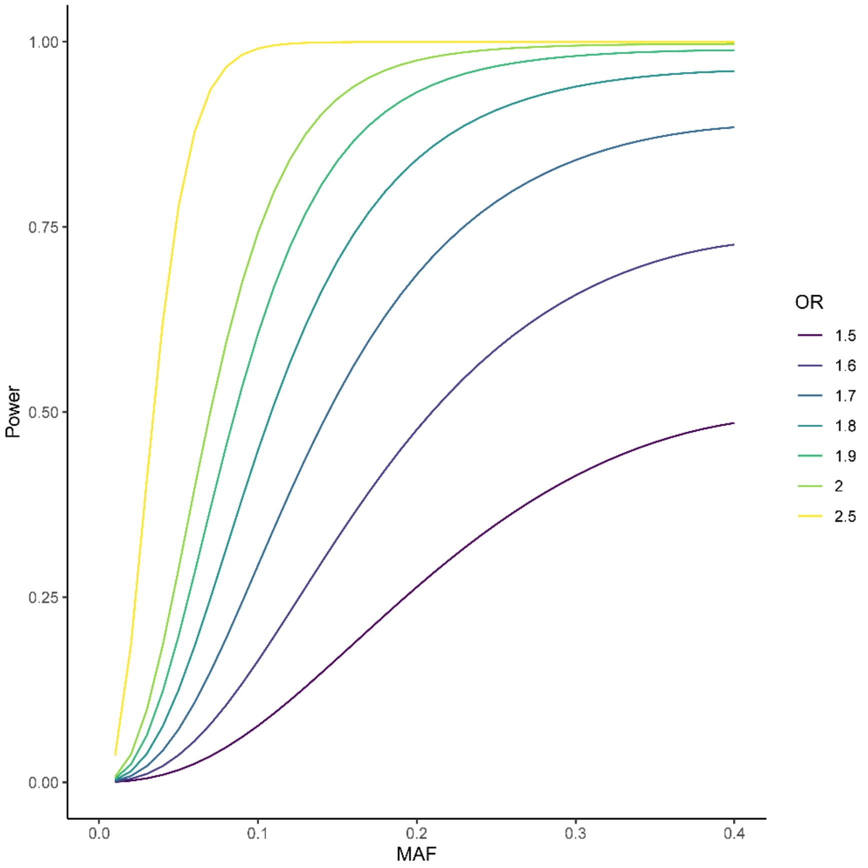

Power estimates are given for a type I error of 5×10^{-5}, a sample of 402 cases and 401 controls and an additive genetic model according to the MAF (*x*-axis) and effect size, as measured by the OR (color-coded curves).

Supplementary Tables

Supplementary Table 1. Genotypic distributions of the two replicated SNPs between cases and controls in the combined cohort

SNP	Chr	Position[a]	Group	Genotype Frequencies (n)[b]		
**rs9814705**	**3**	**145680345**		**T/T**	**T/C**	**C/C**
			**Cases**	0.678 (578)	0.296 (252)	0.026 (22)
			**Controls**	0.804 (514)	0.185 (118)	0.011 (7)
**rs76647377**	**6**	**985196**		**G/G**	**G/A**	**A/A**
			**Cases**	0.968 (827)	0.032 (27)	0 (0)
			**Controls**	0.909 (582)	0.089 (57)	0.002 (1)

[a] GRCh37.p13
[b] Missing Sanger sequencing data for rs9814705 (143 samples) were obtained by best-guess imputation.
