# Supplementary material for: Genome-wide association study of Buruli ulcer in rural Benin highlights role of two LncRNAs and the autophagy pathway
Source: Commun Biol. 2020 Apr 20;3:177. doi: 10.1038/s42003-020-0920-6 (PMC7171125; doi:10.1038/s42003-020-0920-6)
Supplement: Supplementary file 5 — Description of Additional Supplementary Files [file 42003_2020_920_MOESM5_ESM.pdf]

Supplementary Data legends:

Supplementary Data 1. Variants sent to replication.
m, minor allele; M, major allele.
MAF, global minor allele frequency.
GLM, generalized linear model
NA stands for not available, corresponding to genotyped variants that did not pass quality control filters.
─ corresponds to variants displaying association in the opposite direction with respect to the association in the discovery cohort, e.g. rs34060873, the best hit using the Cox model in the GWAS sample is likely a false positive.
[a] Annotation from Variant Effect Predictor and SNPnexus.
[b] The distance from the closest gene is given in bases.
[c] Effects correspond to odds ratios (95% confidence interval) when the GLM model is considered, hazard ratios (95% confidence interval) when the Cox model is considered. The genetic model in defined on the basis of the minor allele.
[d] Unilateral P values in the replication cohort (n=693) were obtained using the same model and method as the one used to identify the variants in the primary cohort. *P* values for SNPs showing association in the opposite direction with respect to the association in the primary cohort were noted "─". *P* values of the two variants showing true replication ($P < 0.01$) are in bold.
[e] We only reported the effects of variants that were in the same direction as the one of the association in the primary cohort.
[f] Variant obtained by imputation.
[g] The other model also displayed P value $< 5\times10^{-5}$ for genotyped variants or *P* value $< 10^{-6}$ for imputed variants

Supplementary Data 2.
Supplementary Data 2a: Association tests for variants located within defensin genes under the additive model with P values < 0.01.
Chr: chromosome; MAF: minor allele frequency. Position in GRCh37.

Supplementary Data 2b: Association tests for eQTL for defensin genes under the additive model with P values < 0.05 in skin sun exposed lower leg and whole blood tissues from the GTEx database.
Chr: chromosome; MAF: minor allele frequency. Position in GRCh37.

Supplementary Data 3.
Supplementary Data 3a: Comparison of the best hits in tuberculosis GWAS recorded in the GWAS Catalog with Buruli ulcer GWAS
Position in GRCh37; Chr: chromosome; NR: not reported

Supplementary Data 3b: Comparison of the best hits in leprosy GWAS recorded in the GWAS Catalog with Buruli ulcer GWAS
Position in GRCh37; Chr: chromosome; NR: not reported
