# Supplementary material for: Genome-wide association study of Buruli ulcer in rural Benin highlights role of two LncRNAs and the autophagy pathway
Source: Commun Biol. 2020 Apr 20;3:177. doi: 10.1038/s42003-020-0920-6 (PMC7171125; doi:10.1038/s42003-020-0920-6)
Supplement: Supplementary file 6 — Reporting Summary [file 42003_2020_920_MOESM6_ESM.pdf]

nature research

Corresponding author(s): Jeremy Manry, Alexandre Alcaïs

Last updated by author(s): Mar 22, 2020

Reporting Summary

Nature Research wishes to improve the reproducibility of the work that we publish. This form provides structure for consistency and transparency in reporting. For further information on Nature Research policies, see Authors & Referees and the Editorial Policy Checklist.

Statistics

For all statistical analyses, confirm that the following items are present in the figure legend, table legend, main text, or Methods section.

n/a	Confirmed	
☐	☒	The exact sample size (n) for each experimental group/condition, given as a discrete number and unit of measurement
☐	☒	A statement on whether measurements were taken from distinct samples or whether the same sample was measured repeatedly
☐	☒	The statistical test(s) used AND whether they are one- or two-sided<br>*Only common tests should be described solely by name; describe more complex techniques in the Methods section.*
☐	☒	A description of all covariates tested
☐	☒	A description of any assumptions or corrections, such as tests of normality and adjustment for multiple comparisons
☐	☒	A full description of the statistical parameters including central tendency (e.g. means) or other basic estimates (e.g. regression coefficient) AND variation (e.g. standard deviation) or associated estimates of uncertainty (e.g. confidence intervals)
☐	☒	For null hypothesis testing, the test statistic (e.g. F, t, r) with confidence intervals, effect sizes, degrees of freedom and P value noted<br>*Give P values as exact values whenever suitable.*
☒	☐	For Bayesian analysis, information on the choice of priors and Markov chain Monte Carlo settings
☒	☐	For hierarchical and complex designs, identification of the appropriate level for tests and full reporting of outcomes
☐	☒	Estimates of effect sizes (e.g. Cohen's d, Pearson's r), indicating how they were calculated

Our web collection on statistics for biologists contains articles on many of the points above.

Software and code

Policy information about availability of computer code

Data collection	Genotypes were assessed with GenTrain implemented in Illumina GenomeStudio software (v2011.1)
Data analysis	All association analyses were performed with R3.6.1 (https://www.r-project.org/), PLINK 1.9, GEMMA, coxme, SNPTEST v2.5.2 64, and ProbABEL software

For manuscripts utilizing custom algorithms or software that are central to the research but not yet described in published literature, software must be made available to editors/reviewers. We strongly encourage code deposition in a community repository (e.g. GitHub). See the Nature Research guidelines for submitting code & software for further information.

Data

Policy information about availability of data

All manuscripts must include a data availability statement. This statement should provide the following information, where applicable:

- Accession codes, unique identifiers, or web links for publicly available datasets
- A list of figures that have associated raw data
- A description of any restrictions on data availability

Association results for the 10,014,109 tested variants are available upon request, as well as the genome-wide variant genotyping and all other relevant data.

Field-specific reporting

Please select the one below that is the best fit for your research. If you are not sure, read the appropriate sections before making your selection.

☒ Life sciences ☐ Behavioural & social sciences ☐ Ecological, evolutionary & environmental sciences

Life sciences study design

All studies must disclose on these points even when the disclosure is negative.

Sample size	We calculated the power of our discovery sample (402 cases and 401 controls) for detecting a significant association with Buruli ulcer coded as a binary trait, assuming a type I error of 5x10-5. Power estimates are shown for an additive model, by MAF, and effect size, as estimated by calculation of the odds ratio (OR) of the tested variants. For example, our sample had 50% power for the detection of variants with an OR of 1.6 and a MAF > 0.2, and 80% power for detecting variants with an OR of 1.8 and a MAF > 0.18. All these power estimates were calculated with QUANTO v1.2.4 (http://biostats.usc.edu/Quanto.html).
Data exclusions	Thirteen samples were excluded from the discovery sample due to a call rate <95% (8 samples), lack of concordance between reported and genetically inferred gender (3 samples), or duplication (2 samples). Fifteen samples were excluded from the replication sample due to a call rate <95% (4 samples), and duplication (11 samples).
Replication	All variants for which evidence for association, in the discovery sample, was obtained with a type I error of less than 5x10-5 (genotyped variants) or 1x10-6 (imputed variants) were retained for genotyping and association testing in the replication sample.
Randomization	DNA preparations from the discovery were randomized in nine 96-well plates for genotyping. The same was done for the replication sample.
Blinding	In the context of our GWAS, blinding was not relevant.

Reporting for specific materials, systems and methods

We require information from authors about some types of materials, experimental systems and methods used in many studies. Here, indicate whether each material, system or method listed is relevant to your study. If you are not sure if a list item applies to your research, read the appropriate section before selecting a response.

Materials & experimental systems

n/a	Involved in the study	
☒	☐	Antibodies
☒	☐	Eukaryotic cell lines
☒	☐	Palaeontology
☒	☐	Animals and other organisms
☐	☒	Human research participants
☒	☐	Clinical data

Methods

n/a	Involved in the study	
☒	☐	ChIP-seq
☒	☐	Flow cytometry
☒	☐	MRI-based neuroimaging

Human research participants

Policy information about studies involving human research participants

Population characteristics	The discovery sample consisted of 408 HIV-free Buruli ulcer cases and 408 exposed controls (354 male and 462 female subjects; the mean ages of the cases and controls were 18.5 and 39.8 years, respectively). We chose to include controls that were older than the cases, as being older meant that they had been exposed to M. ulcerans for a longer time but had remained Buruli ulcer-free). The vast majority of cases were confirmed through the detection of M. ulcerans DNA by polymerase chain reaction (PCR) targeting the insertion element IS2404 which is the most sensitive and specific laboratory test currently available for the diagnostic of Buruli ulcer. Overall, 98.3% of cases in the discovery sample and 45% in the replication sample were laboratory-confirmed through PCR and/or culture examination. All the cases that were not laboratory confirmed were classified as highly probable on the basis of stringent clinical criteria as defined by the World Health Organization.
Recruitment	Between July 2003 and June 2012, 1,524 individuals from the CDTLUB in Pobè, Benin, were enrolled. All participants were living in villages distributed over the Ouémé and Plateau départements (an administrative area equivalent to a county) in an area in which Buruli ulcer was endemic.
Ethics oversight	This study on human genetic susceptibility to Buruli ulcer was approved by the Ethics Committee of the CDTLUB (Pobè, Benin), the International Review Board of the Ministry of Health in Benin (IRB00006860), and the Ethics Committee of the University Hospital of Angers, France (Comité d'Ethique du CHU d'Angers). Written informed consent was obtained from all adult participants. Parents or guardians provided informed consent on behalf of all minors participating in the study.

Note that full information on the approval of the study protocol must also be provided in the manuscript.
